# Cognitive factors contribute to speech perception in cochlear-implant users and age-matched normal-hearing listeners under vocoded conditions

**DOI:** 10.1121/1.5116009

**Published:** 2019-07-17

**Authors:** Erin R. O'Neill, Heather A. Kreft, Andrew J. Oxenham

**Affiliations:** Department of Psychology, University of Minnesota, Elliott Hall, 75 East River Parkway, Minneapolis, Minnesota 55455, USA

## Abstract

This study examined the contribution of perceptual and cognitive factors to speech-perception abilities in cochlear-implant (CI) users. Thirty CI users were tested on word intelligibility in sentences with and without semantic context, presented in quiet and in noise. Performance was compared with measures of spectral-ripple detection and discrimination, thought to reflect peripheral processing, as well as with cognitive measures of working memory and non-verbal intelligence. Thirty age-matched and thirty younger normal-hearing (NH) adults also participated, listening via tone-excited vocoders, adjusted to produce mean performance for speech in noise comparable to that of the CI group. Results suggest that CI users may rely more heavily on semantic context than younger or older NH listeners, and that non-auditory working memory explains significant variance in the CI and age-matched NH groups. Between-subject variability in spectral-ripple detection thresholds was similar across groups, despite the spectral resolution for all NH listeners being limited by the same vocoder, whereas speech perception scores were more variable between CI users than between NH listeners. The results highlight the potential importance of central factors in explaining individual differences in CI users and question the extent to which standard measures of spectral resolution in CIs reflect purely peripheral processing.

## I. INTRODUCTION

The cochlear implant (CI) has been a beneficial and often life-changing treatment for individuals with profound sensorineural hearing loss. However, despite advances in processing strategies, current-steering options, electrode arrays, and surgical techniques, clinicians continue to see a very wide range of outcomes for individual CI users. Many studies exploring the speech-perception abilities of CI users have shown word and sentence recognition rates ranging from 0 to 100% across individuals on any given task (Hast *et al.*, 2015; Lenarz *et al.*, 2012; Mahmoud and Ruckenstein, 2014). Individual patient factors, such as CI experience, age at implantation, duration of deafness, and etiology, have been shown to account for very little variance in performance (less than 10%) in large samples of CI users (Blamey *et al.*, 1996; Blamey *et al.*, 2013). A number of studies have explored the association between cross-modal plasticity and CI outcomes with some finding these changes to be adaptive (Anderson *et al.*, 2017; Rouger *et al.*, 2012; Strelnikov *et al.*, 2013) and others finding them maladaptive (e.g., Lee *et al.*, 2003; Sandmann *et al.*, 2012; Zhou *et al.*, 2018). Anatomical, physiological, and surgical factors that may influence spectral resolution and signal quality, such as etiology of hearing loss, degree of neural survival, electrode-neural distance, and insertion depth of the electrode array, have been studied extensively. While some studies have shown that specific surgical factors account for some variance in hearing outcomes (Aschendorff *et al.*, 2007; Finley *et al.*, 2008; Holden *et al.*, 2013; O'Connell *et al.*, 2016; Skinner *et al.*, 2007; Wanna *et al.*, 2014), other studies have not (Van Der Marel *et al.*, 2015).

The limited number of electrodes and the effects of electrical field spread, due to the distance between the electrode and the spiral ganglion cells, lead to limited spectral resolution in CIs. It has often been shown that speech perception, particularly in noise, is strongly influenced by spectral resolution (Dorman *et al.*, 1998; Friesen *et al.*, 2001). Perhaps because differences in spectral resolution between CI users can be complex in nature and measures of spectral resolution are limited in their sensitivity, this relationship has not always been clear at the level of individual CI users. Although some studies in CI users have found a correlation between measures of spectral resolution (e.g., spectral-ripple discrimination or spatial tuning curves) and speech perception in quiet (Anderson *et al.*, 2011; Henry and Turner, 2003; Henry *et al.*, 2005) and in noise (Gifford *et al.*, 2018; Won *et al.*, 2007), others have not (Anderson *et al.*, 2012). These apparent discrepancies may be in part because most measures of spectral resolution, such as spectral-ripple detection (Anderson *et al.*, 2012; Gifford *et al.*, 2018), spectral-ripple discrimination (Anderson *et al.*, 2011; Anderson *et al.*, 2012; Henry and Turner, 2003; Henry *et al.*, 2005; Won *et al.*, 2007), and spectrotemporal-modulation detection (Aronoff and Landsberger, 2013; Choi *et al.*, 2018; Won *et al.*, 2015), use broadband stimuli, but can be performed using only a limited portion of the entire spectrum (e.g., Anderson *et al.*, 2011). In contrast, speech perception benefits from information across the entire spectrum, meaning that good spectral resolution at just one cochlear location will not be sufficient to provide good intelligibility. Also, although a recent large-scale study by Gifford *et al.* (2018) showed a positive correlation between spectral-modulation detection thresholds and sentence perception, the variability around the trendline was very large, making it difficult to predict speech performance on an individual basis.

The field of CI research has focused primarily on possible peripheral differences between users, with less attention being paid to differences at higher levels of processing. Studies with hearing-aid (HA) users have pointed to working memory and various cognitive abilities as possible factors contributing to hearing outcomes, with better working memory and cognitive abilities associated with better speech perception (Akeroyd, 2008; Arehart *et al.*, 2013; Lunner, 2003; Rönnberg *et al.*, 2013). A large-scale study of older adults with hearing loss also found that visual measures of cognitive-linguistic processing and auditory measures of environmental sound identification accounted for the most variance in aided speech understanding (Humes *et al.*, 2013). However, the correlations between cognitive factors and speech perception have been less clear in studies with adult CI users. A recent study of CI users by Moberly *et al.* (2017) found correlations between speech perception in noise and cognitive control, as well as with auditory but not visual working memory. Heydebrand *et al.* (2007) also found that better verbal working memory was associated with improvement in word recognition six months after CI activation, but general cognitive ability and processing speed were not. In the same vein, Hua *et al.* (2017) found correlations between some, but not all, measures of cognitive skills and working memory and the perception of words and sentences in quiet and in noise in bimodal listeners (those with a CI in one ear and a HA in the other).

In lieu of exploring correlations with cognitive measures, other researchers have looked at the use of semantic context as a way to tap into “top-down” processes that may affect speech perception in hearing-impaired listeners. It is known that semantic context is leveraged in commonly encountered acoustic environments (e.g., Baskent *et al.*, 2016; Bhargava *et al.*, 2014; Marslen-Wilson and Welsh, 1978; Signoret *et al.*, 2018), and it may be that CI users rely more heavily on such context to compensate for the degraded input. One study with older hearing-impaired adults suggested that they rely more heavily on semantic context than older adults with normal hearing when performing speech-perception tasks, with the perception of low-context or semantically anomalous sentences requiring more cognitive effort (Moradi *et al.*, 2014). Recent work by Winn (2016) used pupillometry measures to demonstrate decreased listening effort for high-predictability versus low-predictability sentences in CI users and normal-hearing (NH) listeners. Interestingly, the effect of sentence predictability on pupil diameter was smaller and occurred later after the stimulus offset in CI users and NH participants listening to vocoded speech than in NH participants listening to unprocessed speech. The pattern of errors shown in CI users also suggested more semantic than phonemic substitutions in cases where participants incorrectly guessed a missed word in a sentence, indicating a heavier reliance on context. Another recent study in CI users found a significant difference in gate size (i.e., duration or proportion of the word presented) required for recognition of the final words of sentences in cases of high versus low expectancy and entropy (Amichetti *et al.*, 2018). Modeling of contextual information in words and sentences by Dingemanse and Goedegebure (2019) has also suggested that CI users make more use of context in speech recognition than NH listeners. Although these studies provide some insight into the use of semantic context in hearing-impaired listeners and CI users, the direct benefit in speech perception for full sentences with semantic context compared to those without has yet to be studied in CI users.

Finally, the individual variability in speech-perception abilities among younger and older NH listeners has rarely been studied under acoustic conditions that are sufficiently degraded to produce average performance similar to that observed in CI users. Thus, it remains unclear how much the use of context information differs, and how much more variable speech perception is between CI users than between NH listeners, under similarly degraded conditions.

In this study, we attempted to assess the relative importance of perceptual and cognitive factors in predicting speech perception in CI users by using a diverse set of psychoacoustic and cognitive measures. The results were compared with those from two different NH control groups: one was age-matched to our CI participants and the other consisted of young NH listeners, mostly undergraduate students, similar to those most commonly used in the comparison groups of earlier studies. The NH participants were presented with materials via a tone-excited vocoder that was designed to simulate the effects of loss of spectral resolution and to produce performance for speech perception in noise that was comparable to that found for the CI users. Speech materials consisted of syntactically correct sentences that were either semantically coherent (context) or incoherent (nonsense), presented both in quiet and in noise. Psychoacoustic measures included broadband spectral-ripple detection and discrimination. All participants also completed two different cognitive tests: a reading-span test, as a measure of verbal (visual) working memory (Conway *et al.*, 2005), and Raven's Advanced Progressive Matrices, as a measure of non-verbal intelligence (Raven *et al.*, 1998).

If variability in speech understanding among CI users is due primarily to individual differences in spectral resolution, then measures of spectral resolution should correlate more strongly with speech perception than the measures of cognitive performance. In contrast, for the NH listeners, where spectral resolution will be limited by the signal processing of the vocoder rather than the individual peripheral auditory systems, it may be that cognitive factors will explain any variance between listeners. In addition, we predicted that better working memory and cognitive function would increase the difference in performance between context and nonsense sentences because working memory is required to take advantage of semantic context (Signoret *et al.*, 2018). Our overall hypothesis was that context sentences may depend more on cognitive function, whereas nonsense sentences may increase reliance on bottom-up processes (Mattys *et al.*, 2005), and may thus be mediated more by peripheral factors, as reflected by our measures of spectral resolution.

## II. GENERAL METHODS

### A. Participants

A total of 30 adult CI users (23 females and 7 males) ranging in age from 20 to 80 years (mean = 61.5 years; standard deviation, SD = 12.8) were tested. All CI users had at least one year of CI use, with experience ranging between 1 and 28 years (mean = 10.4 years; SD = 7.3). The duration of hearing loss prior to implantation varied between CI users from less than a year to 41 years (mean = 10.2 years; SD = 10.8). Twenty-two of the CI users used Advanced Bionics devices, five used Cochlear devices, and three used Med-El devices. All CI users were post-lingually deafened, with the exception of one CI user who was deafened peri-lingually, followed by immediate implantation and strictly oral instruction. A group of 30 NH adults (23 females and 7 males), age-matched to the CI group with ages ranging from 20 to 78 years (mean = 61.5; SD = 12.7) were also tested. An additional control group was tested, consisting of 30 NH young adults (21 females and 9 males) ranging in age from 18 to 30 years (mean = 20.6; SD = 2.5). All participants were native speakers of American English. Normal hearing for the young listeners was defined as having pure-tone audiometric thresholds less than 20 dB hearing level (HL) at all octave frequencies between 250 and 8000 Hz with no reported history of hearing disorders. Normal hearing for the age-matched listeners was defined as having pure-tone audiometric thresholds less than 20 dB HL at all octave frequencies between 250 and 2000 Hz and no more than 30 dB HL at 4000 and 6000 Hz, with no reported history of hearing disorders. Since close age-matching with the CI group was a priority, this audiometric criteria was a compromise that allowed us to successfully recruit older participants with relatively normal hearing. The average threshold for age-matched listeners at 8000 Hz was 22 dB HL (SD = 15), with individual thresholds ranging from 0 to 60 dB HL.

All the CI users listened with one CI. Bilateral CI users were instructed to use whichever processor they thought gave them better speech perception and to remove the other processor. Four unilateral CI users with some residual hearing in their contralateral ear were instructed to remove hearing aids and to insert an ear plug in the non-CI ear, which was worn for the entirety of the experiment. No CI user with residual hearing had unaided audiometric thresholds better than 70 dB HL at any frequency, which should have rendered any of our stimuli inaudible, particularly after the 10–15 dB of attenuation expected by the ear plug. All four participants with residual hearing self-reported that their CI ear was better for speech perception than their HA ear. All CI users were asked to use processor settings (volume, sensitivity, program, noise reduction, directional microphones) typical of their everyday use.

All experimental protocols were approved by the Institutional Review Board of the University of Minnesota, and all listeners provided informed written consent prior to participating. The same 90 participants completed all experiments in this study.

### B. Order of experiments

The experiments in this study were completed in the order in which they appear: speech perception was completed first (context then nonsense sentences), followed by the cognitive measures (working memory then non-verbal intelligence), and finally measures of spectral resolution (spectral-ripple discrimination then detection). Generally, participants completed all testing in two 2-h sessions within a two-week period. All speech-perception testing was completed in the first session and measures of cognition and spectral resolution were completed in the second session. A small number of participants (five CI users, six age-matched NH listeners, and two young NH listeners) completed the testing in three sessions due to lack of schedule flexibility and differences in testing pace.

## III. EXPERIMENT 1: SPEECH PERCEPTION WITH CONTEXT AND NONSENSE SENTENCES

### A. Stimuli

#### 1. Context sentences

The context speech materials were sentences taken from the Harvard-IEEE speech corpus (Rothauser *et al.*, 1969), recorded by a single female talker. An example of a context sentences is “A *rod* is *used* to *catch pink salmon*,” with keywords in italics. Each context sentence contained five keywords and there were ten sentences per list.

#### 2. Nonsense sentences

The nonsense speech materials were sentences taken from the Helfer (1997) lists of nonsense sentences, recorded by a different single female talker, as used by Freyman *et al.* (2013) and Ruggles *et al.* (2014), among others. The nonsense sentences were created using common one- and two-syllable nouns and verbs taken from the Thorndike-Lorge lists of most common words (Thorndike and Lorge, 1945), with each sentence containing three keywords and between five and seven total words. Each nonsense sentence was constructed either in the form, article *noun* (auxillary verb) *verb* (preposition) article *noun*, or *verb* article *noun* preposition article *noun*, where italicized words are key words and items in parentheses occur in some, but not all, sentences. An example of a nonsense sentence was “A *shop* can *frame* a *dog*,” with keywords in italics. Each nonsense sentence contained three keywords and there were ten sentences per list.

#### 3. Signal processing

Both context and nonsense sentences were presented in quiet and in Gaussian noise, spectrally shaped to match the long-term spectrum of each speech corpus. The speech and noise were mixed at the appropriate signal-to-noise ratio (SNR) before further processing and presentation to the participants. For the NH listeners, the mixture was passed through a 16-channel tone-excited vocoder with the center frequencies taken from the Advanced Bionics standard clinical map. For the CI users, the stimuli were divided into subbands based on each individual CI user's device and clinical map. The number of channels ranged from 7 to 22, depending on the type of CI processor and the number of deactivated electrodes in each CI.

The bandpass filters used to generate the subbands were high-order (947) finite impulse response (FIR) filters, generated with the *fir1* function in matlab (Mathworks, Natick, MA), producing very little overlap between the spectral content of adjacent subbands and a flat frequency response (±0.05 dB) within the entire passband. The impulse responses from the linear-phase filters were time-aligned, reaching their peaks at a delay of approximately 20 ms, independent of filter center frequency. The temporal envelope from each subband was then extracted using a Hilbert transform, and the resulting envelope was lowpass filtered using a fourth-order Butterworth filter with a cutoff frequency of 50 Hz. This cutoff frequency was chosen to reduce possible voicing periodicity cues and to reduce the possibility (for NH listeners) that the vocoder produced spectrally resolved components via the amplitude modulation of the tonal carriers. The resulting temporal envelopes were used to modulate pure-tone carriers with frequencies corresponding to the center frequencies of each channel, which were then presented to the CI users. Stimuli were processed this way for the CI users to maintain consistency in vocoder processing among groups of listeners, so that the filtered envelopes were lowpass filtered at 50 Hz for all conditions and groups. A previous study showed no performance differences in CI users when comparing similarly vocoded to unprocessed stimuli (Oxenham and Kreft, 2014). For the NH listeners, the effects of current spread were simulated via the vocoder by modulating each carrier by the weighted sum of the intensity envelopes from all 16 channels (Oxenham and Kreft, 2014). The weights used in this sum were selected to produce attenuation slopes of 12 dB/octave on either side of the center frequency, to simulate sufficient spectral smearing for speech perception to approximate the average performance of CI users.

The speech was adjusted to a root-mean-square (rms) level of 65 dB sound pressure level (SPL), as measured at the participant's head, and the noise level was adjusted to produce the desired SNR. The noise was gated on 1 s before the beginning of each sentence and gated off 1 s after the end of each sentence. The SNRs were selected in advance, based on previous studies (Oxenham and Kreft, 2014), to avoid ceiling and floor effects in performance, and were the same for both the context and the nonsense sentences, to facilitate direct comparisons between the two speech corpora.

### B. Procedure

The stimuli were generated using matlab and converted via a 24-bit digital-to-analog converter (L22, LynxStudio, Costa Mesa, CA) at a sampling rate of 22050 Hz. The sounds were presented in a single-walled, sound-attenuating booth located in a quiet room via an amplifier and a single loudspeaker, placed approximately 1 m from the listener at 0° azimuth.

Listeners responded to sentences by typing what they heard on a computer keyboard. One of the oldest CI users did not have adequate typing proficiency to enter responses via a keyboard and so instead spoke the responses into a lapel microphone. The spoken answers were recorded and stored as Windows Media Audio (WMA) files and were later listened to and scored offline. Participants were encouraged to guess individual words, even if they had not heard or understood the entire sentence. Instructions were given orally and participants were asked if they had any questions about procedures before beginning the task. Sentences were scored for keywords correct as a proportion of the total number of keywords presented. Initial scoring was automatic, with each error then checked manually for potential spelling errors or homophones (e.g., wait and weight), which were marked as correct. Before the actual experiment took place, NH listeners were presented with two sentence lists of 20 sentences each from the AzBio speech corpus (Spahr *et al.*, 2012) to acclimate them to the vocoded stimuli before the scored sentences were presented. During this training phase, each sentence was presented visually on the computer screen while the audio was played from the speaker. Participants were instructed to listen to each sentence and try to mentally map what they were hearing with the actual words of the sentence presented on the screen, similar to the procedure used by Litvak *et al.* (2007). The listeners did not type any responses during this phase. This training phase was included to acclimatize NH listeners to the vocoded speech and avoid data contamination due to its initial novelty (McGettigan *et al.*, 2008; O'Neill *et al.*, 2019). Following this training phase, but before the actual testing began, both CI and NH participants completed four lists of each sentence corpus (context and nonsense) in quiet to become comfortable with the procedure.

In the actual experiment, two lists of ten sentences each were completed for each SNR for the context sentences and three lists of ten sentences each were completed for each SNR for the nonsense sentences. This design allowed for a comparable number of keywords to be tested from each corpus; because each context sentence contained five keywords and each nonsense sentence contained only three keywords, each participant was presented with 100 keywords per SNR for the context sentences and 90 keywords per SNR for the nonsense sentences. Every participant completed the context sentences first, followed by the nonsense sentences. After the training block of four lists of ten sentences in quiet, the sentences for each scored condition were presented first at an SNR of 20 dB and then at decreasing SNRs in 5-dB steps to a final SNR of 0 dB. Scored sentences presented in quiet were completed as the final block of each condition. The proportions of correct words in each condition were converted to rationalized arcsine units (RAU) (Studebaker, 1985) before statistical analysis, to mitigate some of the potential effects of floor or ceiling performance.

### C. Results

**FIG. 1.**
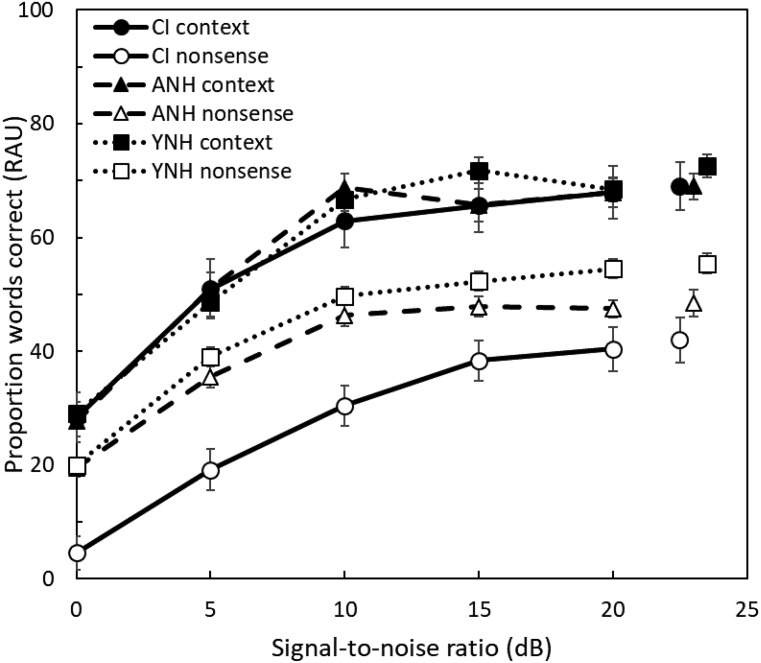
Speech perception for CI users and both groups of NH listeners. The RAU-transformed proportion of keywords from sentences reported correctly is plotted as a function of signal-to-noise ratio for both context (IEEE) and nonsense sentences. Error bars represent ±1 standard error of the mean between listeners.

The mean RAU-transformed proportion of correct keywords from both context and nonsense sentences for the CI group, the age-matched NH group, and the young NH group are shown in Fig. 1. Mean performance in any group always fell between 10 and 73, meaning that the RAU transform (which has the greatest effects at values close to 0 and 100%) did not substantively affect the statistical conclusions. The scores from the context sentences and nonsense sentences are denoted by filled and open symbols, respectively. As expected, performance was similar across all groups for the context sentences, confirming that the 16-channel vocoder with 12 dB/octave spread produces speech perception in NH listeners (in quiet and in noise) that is comparable to that of CI users. Performance for all groups was also poorer for the nonsense than for the context sentences and worsened with decreasing SNR, as expected. Age did not seem to affect performance, as data from the two NH groups were similar across all conditions. Most interestingly, the CI users' performance in the nonsense sentences appeared to be poorer than that of the other two groups.

A repeated-measures analysis of variance (ANOVA) on the RAU-transformed data, with sentence material (context vs nonsense) and SNR as within-subjects factors and group as a between-subjects factor, confirmed a significant main effect of sentence material [*F*(1,87) = 541.3, *P* < 0.001, η_p_^2^ = 0.862], SNR [*F*(5,435) = 744.4, *P* < 0.001, η_p_^2^ = 0.895], and group [*F*(2,87) = 3.2, *P* = 0.046, η_p_^2^ = 0.068]. There were also significant interactions between group and sentence material [*F*(2,87) = 23.97, *P* < 0.001, η_p_^2^ = 0.355], between group and SNR [*F*(10 435) = 3.07, *P* = 0.002, η_p_^2^ = 0.066], and between SNR and sentence material [*F*(5435) = 11.64, *P* < 0.001, η_p_^2^ = 0.118]. The three-way interaction between sentence material, SNR, and group was also significant [*F*(10 435) = 2.94, *P* = 0.001, η_p_^2^ = 0.063].

To further examine these interactions, a series of pairwise comparisons with Bonferroni correction for multiple comparisons was performed. A pairwise comparison between sentence material and group (corrected α = 0.025) showed no significant effect of group for context sentences [*F*(2,87) = 0.12, *P* = 0.89], but did show a significant effect of group for nonsense sentences [*F*(2,87) = 12.76, *P* < 0.001]. Thus, performance was similar between groups for the sentences with context but differed across groups when context was absent. Specifically, performance for the nonsense sentences was significantly poorer for the CI group when compared to both age-matched (*P* = 0.001) and young (*P* < 0.001) NH groups (corrected α = 0.008). However, performance on nonsense sentences did not differ significantly between young and age-matched NH listeners (*P* = 0.189). Pairwise comparisons also confirmed that the effect of sentence material was significant for all three groups [CI group: *F*(1,87) = 359.1, *P* < 0.001; age-matched NH group: *F*(1,87) = 137.77, *P* < 0.001; young NH group: *F*(1,87) = 92.34, *P* < 0.001; corrected α = 0.017], reflecting poorer performance for all groups on sentences without context when compared to those with context.

Other pairwise comparisons examining the interaction between sentence material, SNR, and group (corrected α = 0.0014) showed a significant difference in performance with nonsense sentences between the CI group and the age-matched NH group at poorer SNRs, but not at more favorable SNRs. For example, the CI group performed significantly more poorly than the age-matched NH group on nonsense sentences at SNRs of 0 (*P* < 0.001) and 5 dB (*P* < 0.001), but performed similarly at 20 dB (*P* = 0.058) and in quiet (*P* = 0.109). Performance with nonsense sentences for the CI group was significantly poorer than for the young NH group at both lower [0 (*P* < 0.001) and 5 dB (*P* < 0.001)] and higher [20 dB (*P* < 0.001)] SNRs, as well as in quiet (*P* = 0.001).

## IV. EXPERIMENT 2: WORKING MEMORY AND NON-VERBAL INTELLIGENCE

### A. Methods

#### 1. Stimuli and procedure

**FIG. 2.**
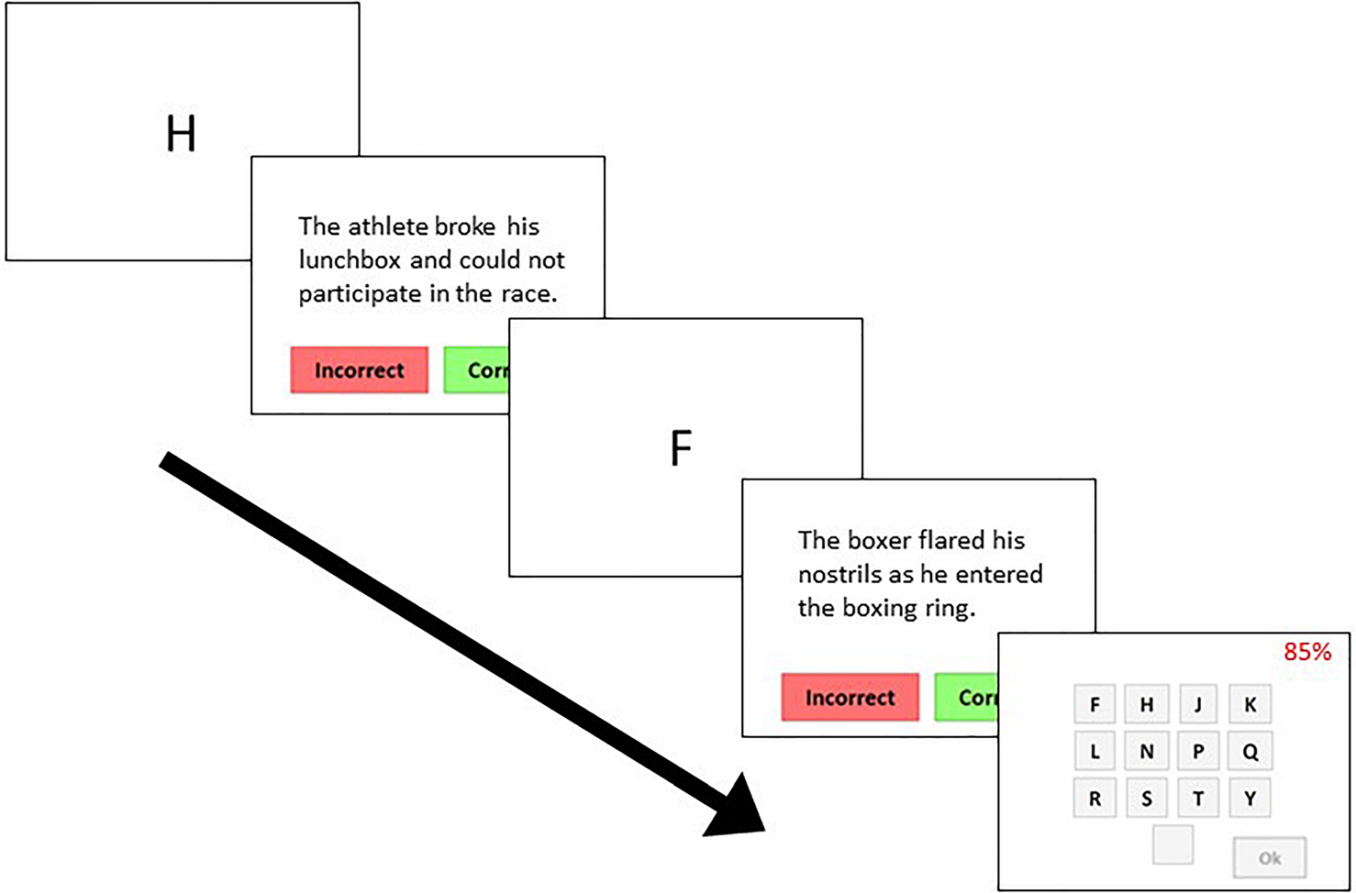
(Color online) Schematic diagram of the reading-span task used to measure visual working memory. The 85% in the upper right corner of the last (front-most) panel represents the minimum percent correct participants had to achieve on sentence decisions by the end of the task for results to be considered valid.

To measure working memory, a reading-span task (Conway *et al.*, 2005) was administered.^1^ The task consisted of individual letters and sentences, presented visually on a computer screen in an alternating fashion. The subset of letters used included F, H, J, K, L, N, P, Q, R, S, T, and Y. Each sentence varied in length from 10 to 15 words and could either be presented as congruent or incongruent contextually. In congruent sentences, all of the words in the sentence adhered to a meaningful context, whereas incongruent sentences contained one word that violated the contextual meaning of the sentence, resulting in a sentence that did not make logical sense, such as “The athlete broke his lunchbox and could not participate in the race.” Each letter was presented for 1 s, followed by a sentence presented for the average amount of time it took each individual participant to judge the correctness of each sentence in an initial practice block. The number of pairs of sentences and letters presented in succession varied from two to seven and was randomized across trials. Each different number of pairs (two through seven) was presented three times for a total of 18 trials. No time limit was imposed while participants had to recall letters seen within a trial and subsequent trials did not initiate until the participant manually moved the procedure forward. A schematic diagram of the procedure is shown in Fig. 2.

All participants completed the reading-span task by following instructions presented visually on a computer screen, while seated alone in a quiet room. Before starting the task, the participants were given an overview of the procedure by an experimenter to avoid any potential confusion and to give the participants an opportunity to ask questions. The participants were instructed to read all the directions presented on the computer screen and were asked to complete a series of training blocks designed to help familiarize them with all parts of the task. The training consisted of three blocks. In the first block, the participants were presented only with letters and were instructed to recall them in the order they were presented. The second block consisted only of sentences during which the participants had to decide whether each sentence was correct or incorrect contextually. The final block alternated the letters and sentences and participants practiced recalling the letters presented while intermittently reading sentences and determining if they were contextually congruent or incongruent. Once the training blocks were complete, the participants were informed that the actual experiment was about to begin.

Each of the 18 blocks of the actual reading-span task consisted of alternating presentations of letters and sentences followed by a screen where participants were asked to recall the letters they saw within that block in the same order they were presented. Participants used a mouse to click on the letters they saw within a given trial on a screen displaying all possible letters (i.e., F, H, J, K, L, N, P, Q, R, S, T, and Y). Each response screen also included a percentage in the upper right corner which represented their overall percent correct on sentence decisions and updated with each successive trial. Participants were told at the start of the test that they had to score at least 85% overall for their scores to be valid upon completion.

Once participants completed the task, letter recall scores were generated, along with the overall percent correct on sentence decisions. A handful of participants did not achieve 85% in their first attempt but, upon re-instruction, maintained this threshold of performance on their second attempt. Two CI users only achieved maximum scores of 84% and 83%, despite multiple repetitions of the task, and reported having difficulty switching between the tasks. Because their performance was close to the cut-off of 85% and because it was clear that the low performance was not because they were strategically ignoring the sentences, the scores from both participants were included in the overall analysis.

The letter recall score, termed the partial-credit unit score, is the percentage of letters correctly recalled in the correct serial position, averaged across trials. For example, if a series of letters presented was L, F, Q, T, K and a participant responded F, L, Q, R, K, they would receive a recall score of 40% on this trial, since only letters Q and K were recalled correctly in the correct serial position. The partial-unit score has been found to be the most reliable and psychometrically sound scoring method for this kind of reading-span task (Conway *et al.*, 2005).

To measure non-verbal intelligence, the paper-and-pencil version of Raven's Advanced Progressive Matrices was used (Raven *et al.*, 1998). The test consisted of 36 matrices, each with eight possible answers. Each matrix problem has eight different combinations of shapes and textures shown in a 3-by-3 grid, with the ninth configuration absent. One of the eight possible answers for each question correctly completes the pattern formed by the combination of shapes in the grid. The test started with easier matrices and became progressively more difficult. Each matrix appeared on a single page in a three-ring binder and participants recorded their answers on a separate sheet of paper. Before beginning the actual test, participants were given two practice matrices by the experimenter. If a participant understood the task and answered both problems correctly, they could begin the actual experiment. If a participant answered the first practice problem incorrectly, the experimenter would walk them through the second practice problem to make sure they understood the task before moving on to the experiment. All participants were given 30 min to complete the experiment and were instructed to answer as many problems correctly as possible in the given time frame. Participants were seated alone at a desk in a quiet room. After 30 min had elapsed, the experimenter returned and instructed participants to put down their pencil and stop taking the test. The test was scored for the total number of correct answers, regardless of the total number of questions answered.

### B. Results

**FIG. 3.**
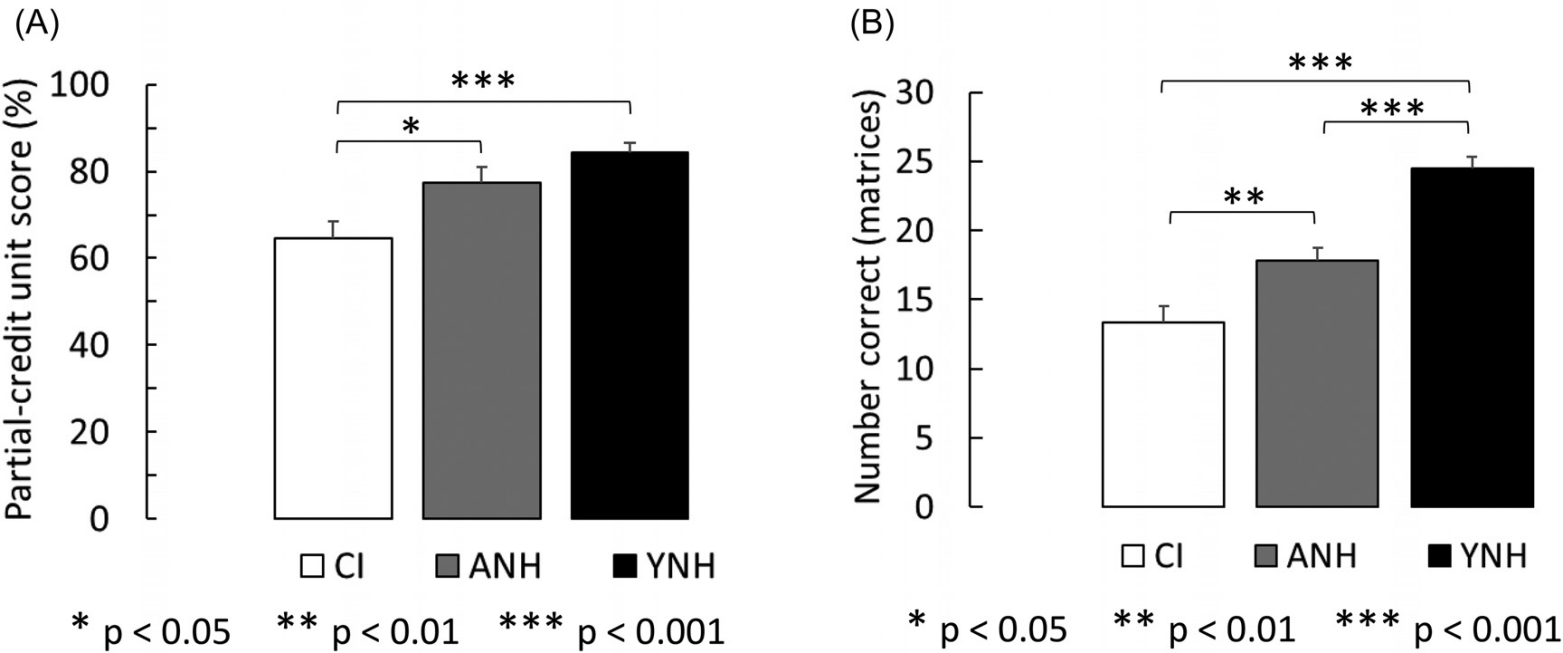
Group-mean scores for working memory and non-verbal intelligence are shown in panels A and B, respectively. Bars in panel A represent mean partial unit scores (proportion of letters within each trial recalled correctly, averaged across trials) on the reading-span task for CI users and both NH groups. Bars in panel B represent the mean number of correctly answered matrix problems (in 30 min) for each group. Error bars represent one standard error of the mean between subjects.

The mean results for the reading-span task from the CI group and both NH groups (age-matched and young) are shown in panel A of Fig. 3. The white, gray, and black bars represent the mean partial unit scores for the CI group, age-matched NH group, and young NH group, respectively. Error bars represent one standard error of the mean between participants. A one-way, between-subjects ANOVA performed on data from all three groups revealed a significant effect of group [*F*(2,87) = 8.93, *P* < 0.001, η_p_^2^ = 0.17]. *Post hoc* comparisons with Bonferroni correction for multiple comparisons (corrected α = 0.017) showed a significant difference between working memory for CI users and both the young (*P* < 0.001) and age-matched (*P* = 0.008) NH groups, but not between age-matched and young NH groups (*P* = 0.146). It is noteworthy that verbal working memory scores on a non-auditory task were significantly poorer for CI users than for both groups of NH listeners. Anecdotally, a number of the CI users who scored lower than average seemed to experience more difficulty with task-switching (between retaining letters and determining sentence coherence) than just with retaining letters in memory. Although we have no direct measure of this difficulty in the current experiment, other studies have shown impairments in cognitive control among older hearing-impaired adults (Lash *et al.*, 2013; Lash and Wingfield, 2014) that may compound deficits in working memory.

The mean results for Raven's Advanced Progressive Matrices are shown in panel B of Fig. 3. The white, gray, and black bars represent data from the CI group, age-matched NH group, and young NH group, respectively. The bars represent the mean number of correctly answered matrix problems (in 30 min) for each group. Error bars represent one standard error of the mean between participants. According to smoothed detailed norms for the U.S. (Raven *et al.*, 1998) on the same test but with no time limit, the young NH group-average score of 24 falls in the 64th percentile for participants aged 18 to 22, perhaps because all participants in this group were university students. The age-matched NH group-average score of 18 falls in the 56th percentile for individuals between the ages of 58 and 62, indicating the group as a whole scored slightly above average. The CI group-average score of 13 represents a score that corresponds to the 32nd percentile for individuals between the ages of 58 and 62. Although these comparisons are not exact, given that a 30-min time limit was imposed on participants in this experiment, population data (Raven *et al.*, 1998) indicate that scores only improve by one, on average, when a time limit is not imposed. Thus, even if group average scores were increased by one for each group, scores for the CI group would still be below the 50th percentile and those for the age-matched NH group would still be above.

A one-way, between-subjects ANOVA on scores from all three groups showed a significant effect of group [*F*(2,87)= 32.94, *P* < 0.001, η_p_^2^ = 0.431]. *Post hoc* comparisons with Bonferroni correction (corrected α = 0.017) revealed a significant difference between CI users and both the age-matched NH (*P* = 0.002) and young NH (*P* < 0.001) groups, as well as between age-matched and young NH listeners (*P* < 0.001). The difference between the CI users and the age-matched NH group on this measure of non-verbal intelligence was not expected, and points to factors other than age that might be influencing speech-perception performance differentially between NH listeners and CI users.

Some studies have found hearing loss to be associated with accelerated cognitive decline in older adults (Claes *et al.*, 2018; Lin *et al.*, 2013; Livingston *et al.*, 2017), which could be reflected in our results. However, since we did not control for socio-economic status, level of education, or sampling bias, and considering the relatively small number of participants in this study, it should not yet be concluded that CI users as a group tend to have lower scores on such measures of non-verbal intelligence.

## V. EXPERIMENT 3: SPECTRAL-RIPPLE DISCRIMINATION AND DETECTION

### A. Methods

#### 1. Stimuli

Spectrally rippled noise was generated using matlab. Gaussian broadband (350–5600 Hz) noise was spectrally modulated, with sinusoidal variations in level (dB) on a log-frequency axis (as in Litvak *et al.*, 2007) using the equation

$$X\left(f\right)=10^{\left(D/2\right)\text{sin}\;\left\{2\pi \left[\;{\text{log}}_{2}\left(f/L\right)\right]f_{s}+\theta \right\}/20},$$

where *X*(*f*) is the amplitude at frequency *f* (in Hz), *D* is the spectral depth or peak-to-valley ratio (in dB), *L* is the lower cut-off frequency of the noise pass band (350 Hz in this case), *f*_*s*_ is the spectral modulation frequency (in ripples per octave), and θ is the starting phase of the ripple function.

The ripple-discrimination task involved spectrally rippled stimuli with a fixed peak-to-valley ratio of 30 dB, while the ripple rate was varied adaptively to track the highest ripple rate or density, in ripples per octave (rpo), at which a phase reversal of the ripples is detectable. This threshold is thought to provide a measure of the limits of spectral resolution, and does not appear to suffer from the potential confounds of temporal-envelope cues, which can affect spectral-ripple detection at high ripple rates (Anderson *et al.*, 2012; Nechaev *et al.*, 2019). The ripple-detection task involved measuring the minimum detectable peak-to-valley ratio at a fixed ripple rate of 0.25, 0.5, 1, or 2 rpo. This task was included because ripple detection thresholds, particularly at low rates (0.25 and 0.5 rpo), have been shown to be correlated to the recognition of speech sounds (Anderson *et al.*, 2012; McKay *et al.*, 2018; Saoji *et al.*, 2009).

The duration of each stimulus was 400 ms, including 20-ms raised-cosine onset and offset ramps. For NH listeners, the stimulus was passed through the same tone-excited envelope vocoder used in Experiment 1, with 16 frequency channels that produced spectral spread equivalent to 12 dB/oct. The CI users were presented with the stimulus unaltered. Both ripple detection and discrimination have been used in previous studies (e.g., Drennan *et al.*, 2014; Henry *et al.*, 2005; Won *et al.*, 2011) and both have been shown to correlate with each other and with more direct measures of spectral resolution, such as spatial tuning curves (Anderson *et al.*, 2011).

The stimuli were presented using the same setup as in Experiment 1, via a loudspeaker positioned at approximately head height and about 1 m from the participant in a single-walled, sound attenuating booth. The average sound level of the noise was set to 60 dBA when measured at the location corresponding to the participant's head. To reduce any possible cues related to overall loudness, the noise level was roved across intervals within each trial by ±3 dB. The starting phase of the spectral modulation was selected at random with uniform distribution for each trial to reduce the potential for any consistent local intensity cues that fixed-phase stimuli might create.

#### 2. Procedure

A three-interval, three-alternative forced-choice procedure was used for both tasks. Correct-answer feedback was provided after each trial. For the ripple-discrimination task, all three intervals contained spectrally rippled noise. In each trial, two intervals contained spectral ripples that had the same starting phase (selected at random from a uniform distribution on each trial), and in the remaining interval the phase of the ripple was reversed (180**°** phase shift). The order of the three intervals (two same, one reversed) was randomized on every trial with equal *a priori* probability. Listeners were instructed to choose the interval that sounded different from the other two. The first trial of each run started at a ripple rate of 0.25 rpo, corresponding to a single ripple across the 4-octave passband. In each successive trial, the ripple rate was varied adaptively using a 2-down, 1-up rule, with rpo initially increasing or decreasing by a factor of 1.41. After the first two reversals, the step size changed to a factor of 1.19 and decreased again to a factor of 1.09 after two more reversals. Each run was considered complete after a total of ten reversals, and the geometric mean ripple rate at the last six reversal points were used to determine the threshold.

For the ripple-detection task, one of the three intervals had a spectral ripple and the other two intervals contained spectrally flat noise. Listeners were instructed to select the interval that sounded different (i.e., the one with spectral modulation). The first trial of each run started at a peak-to-valley ratio of 20 dB. In each successive trial, the ripple depth was varied adaptively using a 2-down, 1-up rule, with the peak-to-valley ratio initially increasing or decreasing by 4 dB. After the first two reversals, the step size changed to 2 dB and decreased again to 0.5 dB after two more reversals. Each run was considered complete after a total of ten reversals, and the mean peak-to-valley ratio at the last six reversal points were used to determine the threshold. The maximum peak-to-valley ratio allowed was set to 50 dB. If participants failed to accurately respond at 50 dB on six trials, the run was terminated and 50 dB was recorded as the ripple detection threshold for that run. This occurred occasionally at the highest ripple rate (2.0 rpo).

All participants completed four runs of the ripple-discrimination task, followed by four runs of each ripple rate (0.25, 0.5, 1.0 and 2.0) in the ripple-detection task. The ripple rates in the ripple-detection task were randomized within blocks, with each block containing each ripple rate once. For each ripple task (discrimination and detection) and condition (different ripple rates in the detection task), the first run for each participant was considered practice, and the last three thresholds were used to compute a mean threshold for each individual participant. Instructions were given orally and participants were asked if they had any questions about procedures before beginning each task.

### B. Results

**FIG. 4.**
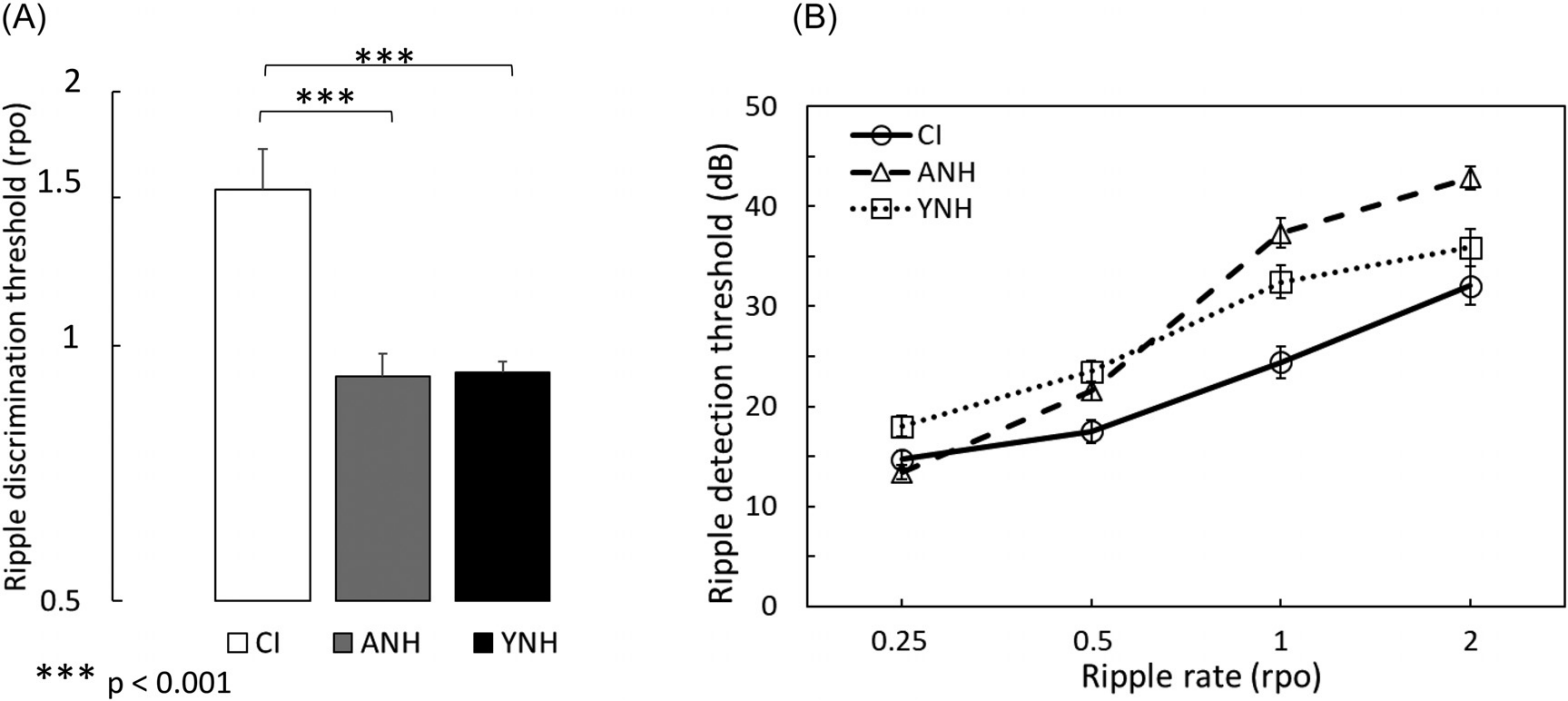
Group means for the ripple discrimination and detection tasks are shown in panels A and B, respectively. Bars in panel A represent mean ripple discrimination thresholds (in ripples per octave) for CI users and both groups of NH listeners. Panel B shows mean ripple-detection thresholds (in dB) for four different ripple rates. Error bars represent one standard error of the mean between subjects.

The mean results for the ripple-discrimination task from the CI group and both NH groups (age-matched and young) are shown in panel A of Fig. 4. The white, gray, and black bars represent the CI group, age-matched NH group and young NH group, respectively. The bars represent the mean ripple discrimination threshold (in rpo) for each group. Error bars represent one standard error of the mean between participants.

Contrary to what might be expected based on results of Experiment 1, spectral discrimination thresholds were better for the CI group than for both NH groups listening through a vocoder with 12 dB/oct spread. Discrimination thresholds from the NH groups were very similar, with the age-matched group averaging 0.92 rpo and the young group averaging 0.93 rpo. The average ripple rate at threshold for the CI group was 1.53 rpo. A one-way, between-subjects ANOVA confirmed a significant effect of group [*F*(2,87)= 15.07, *P* < 0.001, η_p_^2^ = 0.257], with *post hoc* comparisons with Bonferroni correction (corrected α = 0.017) showing no significant difference between NH groups (*P* = 0.894), but a significant difference between thresholds for CI users and both age-matched (*P* < 0.001) and young (*P* < 0.001) NH groups. No differences were observed in thresholds of CI users with different processing strategies (e.g., n-of-m vs continuous interleaved sampling, CIS), although the number of participants using each strategy was too small to perform a formal statistical analysis.

The mean results from all three groups for the ripple-detection task are shown in panel B of Fig. 4. Results from each group are shown with different symbols, with data from the CI group in circles, the age-matched NH group in triangles, and the young NH group in squares. The symbols represent mean ripple-detection thresholds in dB for four different ripple rates, including 0.25, 0.5, 1.0, and 2.0 rpo. Consistent with results from the ripple-discrimination task, CI users had lower (better) ripple-detection thresholds than young NH listeners at all ripple rates. Results from young NH listeners follow a similar trend to that of CI users, with detection thresholds increasing with increasing ripple rate, resulting in a difference in thresholds of about 17 dB between the 0.25 rpo and the 2.0 rpo conditions. However, results from the age-matched NH group differ somewhat from both the CI group and young NH group, with thresholds increasing more rapidly with increasing ripple rate. The mean threshold for the age-matched NH group at 0.25 rpo is about 1 dB lower than the mean threshold for the CI group at the same rate but almost 11 dB higher than for the CI group at 2.0 rpo. The difference in average threshold between the lowest (0.25) and highest (2.0) ripple rates tested is about 30 dB, which is almost double the difference in thresholds found for the other two groups. In fact, this difference may be underestimated, since 24 age-matched NH participants were not able to reliably detect the ripples at 2 rpo on at least one of three averaged runs, even when the ripple depth was set at its maximum of 50 dB. Only ten of the CI users and 16 of the younger NH listeners were not able to detect the ripple under the same conditions.

A repeated-measures ANOVA on the detection thresholds with ripple rate as a within-subjects factor and group as a between-subjects factor revealed a significant effect of ripple rate [*F*(3,261) = 329.18, *P* < 0.001, η_p_^2^ = 0.791], a significant effect of group [*F*(2,87) = 10.54, *P* < 0.001, η_p_^2^ = 0.195] and a significant interaction between ripple rate and group [*F*(6,261) = 14.70, *P* < 0.001, η_p_^2^ = 0.253]. *Post hoc* comparisons with Bonferroni correction for multiple comparisons (corrected α = 0.0042) showed that detection thresholds for the CI group and age-matched NH group were not significantly different at lower ripple rates [0.25 rpo (*P* = 0.29) and 0.5 rpo (*P* = 0.008)], but were significantly different at higher ripple rates [1.0 rpo (*P* < 0.001) and 2.0 rpo (*P* < 0.001)]. Thresholds for the young NH group were not significantly different from the CI group at the highest [2.0 rpo (*P* = 0.112)] and lowest ripple rates [0.25 rpo (*P* = 0.009)], but were significantly different at ripple rates of 0.5 (*P* < 0.001) and 1.0 (*P* = 0.001) rpo.

## VI. INDIVIDUAL DIFFERENCES: VARIANCE WITHIN AND CORRELATIONS BETWEEN MEASURES

### A. Comparing within-group variances

To compare the amount of variance in each experimental measure for the CI group and both NH groups, the factor by which the variance was greater between each pair of groups was calculated and Levene's Test for Equality of Variances was performed. Results from these comparisons are shown in Table I. The CI users had significantly greater between-subject variance on all four speech measures (context and nonsense sentences in quiet and in noise) compared to the age-matched NH group and young NH group. It should be noted that the magnitude by which the variance was greater for the CI group when compared to the young NH group was larger than when compared to the age-matched NH group, suggesting more variance among older participants overall. However, the variance for the age-matched NH group was not significantly greater than the young NH group on any of the speech perception measures. Interestingly, CI users did not show significantly more between-subject variance on the ripple-detection task than the age-matched or young NH listeners. The CI group did show significantly more variance on the ripple-discrimination task, although average thresholds for this measure were also quantitatively different from those of both NH groups, with the average for CI users being significantly better. On the ripple-detection task, however, thresholds for CI users were much more similar to those of the NH groups and did not show increased variance.

**TABLE I.** Factor by which the variance was greater for the first group (listed) than the second group for different measures. The groups were the CI users (CI), the young NH listeners (YNH), and the age-matched NH listeners (ANH). Levene's Test for Equality of Variances was also performed for each comparison to calculate which differences were significant. *p < 0.05. **p < 0.01. ***p < 0.001.

Task	CI vs YNH	CI vs ANH	ANH vs YNH
Context quiet	4.34**	3.41*	1.27
Context noise average	6.14***	3.69**	1.66
Nonsense quiet	5.17***	2.89**	1.79
Nonsense noise average	6.55***	4.59***	1.43
Working memory	2.95***	1.10	2.67
Non-verbal intelligence	1.91	1.56	1.22
Ripple discrimination	5.07***	4.58**	1.11
Ripple detection average	1.30	1.82	0.72

Another noteworthy difference in variance between groups was that the CI group had significantly more variance on the reading-span task, measuring working memory, than the young NH group but not the age-matched NH group. If working memory does indeed play a significant role in performance for CI users, this increase in variability when compared to young NH listeners further supports the use of age-matched controls in studies of CI users.

### B. Correlations between measures of speech perception, spectral resolution, and cognitive function

The possible influence of both peripheral and central factors on speech-perception performance was explored by correlating speech perception scores with the measures of spectral resolution and cognitive function. Because of the large number of potential correlations, the correlations were restricted to those based on *a priori* hypotheses for CI users (e.g., the relationship between speech perception and spectral resolution) and on the findings from an exploratory principal component analysis (PCA) of the data. The factors included in the PCA analysis were context and nonsense speech perception, working memory, non-verbal intelligence, spectral-ripple discrimination and detection, age, and years of CI use (for the CI group only). Pearson's *r* values and corresponding *P* values for various comparisons, selected based on our initial hypotheses and on the PCA loadings, are shown in Table II. To summarize speech performance, scores were averaged for each participant across all SNRs (including quiet) to produce an overall speech score for each type of speech material (context and nonsense). Ripple-detection thresholds were also averaged across ripple rates where thresholds for all groups were above floor performance (i.e., 0.25, 0.5, and 1.0 ripples/octave).

**TABLE II.** Correlations between experimental measures for CI users and both NH groups. Scores on context and nonsense sentences are averaged for a general speech perception measure in the bottom half of the table. Pearson's *r* values that have *p*-values less than 0.05 are bolded to highlight significant correlations.

Correlations between experimental measures	CI		ANH		YNH	
	*R*	*p*-value	*R*	*p*-value	*R*	*p*-value
Context sentences vs nonsense sentences	**0.887**	<**0.001**	**0.847**	<**0.001**	**0.865**	<**0.001**
Ripple discrimination vs ripple detection	**−0.826**	<**0.001**	**−0.648**	<**0.001**	**−0.563**	**0.001**
Working memory vs non-verbal intelligence	**0.417**	**0.022**	**0.712**	<**0.001**	**0.664**	<**0.001**
Speech perception vs ripple discrimination	**0.556**	**0.001**	0.340	0.066	0.315	0.090
Speech perception vs ripple detection	**−0.529**	**0.003**	**−0.514**	**0.004**	−0.273	0.144
Speech perception vs working memory	**0.430**	**0.018**	**0.447**	**0.013**	0.104	0.585
Speech perception vs non-verbal intelligence	0.319	0.086	**0.585**	**0.001**	0.198	0.293
Ripple discrimination vs working memory	0.168	0.374	**0.525**	**0.003**	**0.552**	**0.002**
Ripple detection vs working memory	−0.217	0.250	**−0.501**	**0.005**	−0.311	0.095
Ripple discrimination vs non-verbal intelligence	0.170	0.369	0.315	0.090	**0.628**	<**0.001**
Ripple detection vs non-verbal intelligence	−0.341	0.065	−0.241	0.199	**−0.435**	**0.016**

Considering first the measures of speech perception, strong correlations were found in all three groups between performance on context sentences and nonsense sentences (CI group: *r* = 0.887, *P* < 0.001; age-matched NH group: *r* = 0.847, *P* < 0.001; young NH group: *r* = 0.865, *P* < 0.001). Perhaps not surprisingly, given these very high correlations, there were no correlations observed between the *difference* in performance between context and nonsense sentences and either of the cognitive measures for any group (*p* > 0.58 in all cases). Because of the high correlations between the two speech measures, we averaged performance on context and nonsense sentences to create a single global measure of speech perception for each participant that was used in the remainder of the correlations.

Significant correlations were observed between the measures of speech perception and spectral resolution. The CI group showed significant correlations when comparing speech perception and ripple-discrimination thresholds (*r* = 0.556, *P* = 0.001) and ripple-detection thresholds (*r* = −0.529, *P* = 0.003). However, the age-matched NH group also showed significant correlations, of roughly the same magnitude, between speech perception and ripple-detection thresholds (*r* = −0.514, *P* = 0.004). The young NH group showed a different trend, with speech perception not significantly correlating with ripple-detection thresholds (*r* = −0.273, *P* = 0.144).

In line with previous research indicating a link between working memory and speech perception for older hearing-impaired listeners (Akeroyd, 2008; Lunner, 2003; Zekveld *et al.*, 2012), speech perception was significantly correlated with scores of verbal working memory for CI users (*r* = 0.430, *P* = 0.018) and age-matched NH listeners (*r* = 0.447, *P* = 0.013), but not for young NH listeners (*r* = 0.104, *P* = 0.585). The significant correlation observed for the CI users and age-matched NH listeners, but not younger NH listeners, suggests that both age and hearing loss affect the impact of working memory on speech perception in noise (Füllgrabe and Rosen, 2016).

Scores on Raven's Advanced Progressive Matrices did not show a significant correlation with speech perception for CI users (*r* = 0.319, *P* = 0.086), indicating non-verbal intelligence was not a strong predictor of speech performance. Interestingly, age-matched NH listeners did show a significant correlation between non-verbal intelligence and speech perception (*r* = 0.585, *P* = 0.001), whereas young NH listeners did not (*r* = 0.198, *P* = 0.283). Overall, no clear picture emerged relating non-verbal intelligence with speech performance across all three groups.

Correlations between speech-perception scores and the cognitive measures for both the CI users and the age-matched NH listeners support suggestions that cognitive factors can affect speech perception (Conway *et al.*, 2014; Heydebrand *et al.*, 2007; Moberly *et al.*, 2017). Interestingly, the proportion of variance accounted for was quite similar to that accounted for by the measures of spectral resolution. The lack of correlation between the spectral resolution and cognitive measures in the CI users suggests that these factors are accounting independently for variance. To pursue this question further, we devised a composite measure of spectral resolution. The composite score was derived by combining the within-group z-scores from the spectral-ripple discrimination thresholds and the average ripple-detection thresholds for ripple rates of 0.25, 0.5, and 1.0 rpo. In addition, a composite measure of cognitive performance was derived, which was the combined within-group z-scores from the two cognitive tests. A multiple linear regression analysis using the CI users' combined speech score as the dependent variable showed that the two composite measures accounted for 41.2% of the total variance. The cognitive and spectral resolution composite measures independently accounted for 23% and 56% of the 41.2% explained variance, respectively.

## VII. DISCUSSION

The aim of this study was to analyze peripheral and cognitive factors that may influence speech perception of CI users, and to compare the results, in terms of overall performance and variability, with those from young and age-matched NH participants, listening through a vocoder designed to similarly limit speech perception. The measures included speech perception with context and nonsense sentences, spectral-ripple detection and discrimination, visual working memory, and non-verbal intelligence. The main findings and their implications are discussed below.

### A. Greater reliance on semantic context for CI users than NH listeners

One striking finding from this study was the large decrement in speech intelligibility found for CI users when semantic context was not available. Whereas the difference in performance of the age-matched and young NH listeners between context and nonsense sentences was about 15 percentage points on average, the difference for the CI users was about 30 percentage points (see Fig. 1). This difference was observed despite the fact that performance in the context sentences was very similar across all three listener groups, due to the use of a vocoder with the NH groups. One interpretation of this finding is that CI users have learned through experience to make more use of semantic context information than NH listeners, due perhaps to the fact that the CI users are continually presented with degraded auditory input. This interpretation is supported by findings from a recent study (Dingemanse and Goedegebure, 2019), showing that CI users made more use of contextual information in recognition of words and sentences than NH listeners. Because the same effects are not observed in NH listeners even when the stimuli are degraded by a vocoder, they may reflect longer-term adaptation of CI users to chronically degraded auditory input by learning to rely more heavily on context (Glick and Sharma, 2017). However, because our two sentence corpora also differed on other dimensions (e.g., different number of keywords, different talkers, etc.), other interpretations remain possible, as discussed in the section on limitations below.

### B. Correlations between measures and variance within measures suggest peripheral and cognitive contributions to speech perception

The large variability in speech perception and spectral resolution within the population of CI users is well documented (Hast *et al.*, 2015; Lenarz *et al.*, 2012; Mahmoud and Ruckenstein, 2014); much less attention has been paid to variability within the NH population when the auditory input is degraded to simulate the average performance of CI users. Our results show much greater variability between CI users in measures of speech perception than between young or age-matched NH listeners, supporting the hypothesis that CI-specific factors (such as the electrode placement or neural survival) underlie a larger proportion of the variance observed. Comparisons of the within-group variances in the measures of spectral resolution were more mixed. The CI group had significantly more variance than the age-matched NH group for the ripple-discrimination thresholds. However, the amount of variance for thresholds averaged across ripple-detection rates was actually similar for the CI and age-matched NH groups.

Spectral ripple-detection thresholds are thought to reflect not only spectral resolution but also intensity resolution, as detection requires the ability to detect the level differences between the spectral peaks and valleys after auditory filtering or CI presentation (Anderson *et al.*, 2012). From past studies, it appears as if ripple detection at low rates (<1 rpo), i.e., those most likely to reflect intensity resolution, are best correlated with speech perception (Anderson *et al.*, 2012; Litvak *et al.*, 2007; McKay *et al.*, 2018; Saoji *et al.*, 2009). A better measure of spectral resolution may therefore be the difference in thresholds between a low rate (i.e., 0.25 rpo) and a high rate (i.e., 1.0 rpo). However, even with this difference measure, the CI group still did not have significantly more variance than either NH group. Finally, the within-group variance in the two cognitive measures was similar between the two age-matched groups and was less among the younger group. Thus, the variance of these cognitive measures did not differ with hearing status, once age was accounted for. Nevertheless, as mentioned in Sec. IV B, mean absolute performance in both tasks was lower among the CI users in this sample than in either NH group.

The strong correlation between the two measures of speech perception (context and nonsense sentences) in all three listener groups was perhaps not surprising, given the similarity in task and materials. Nevertheless, the results do not support our initial hypothesis that working memory and/or non-verbal intelligence may be more related to performance in context sentences, whereas measures of spectral resolution may be more related to performance in nonsense sentences. It is possible that understanding nonsense sentences required more cognitive resources than we initially predicted, and that working memory and/or non-verbal intelligence may have mediated performance on both context and nonsense sentences. This interpretation seems plausible given the strong correlation between the two measures, even for the NH groups.

Correlations between the speech measures and the measures of spectral resolution were relatively high among the CI users (*r* ≈ 0.5), confirming the relationship between measures of spectral resolution and speech perception that has been found in many other studies (Henry *et al.*, 2005; Holden *et al.*, 2016; Jeon *et al.*, 2015; Won *et al.*, 2011; Zhou, 2017). However, the similarly high correlations (*r* = −0.514) between spectral-ripple detection and speech perception in the age-matched NH higher listeners was puzzling, as spectral resolution in that group was limited by the vocoder, and thus should have been similar for all NH listeners. Again, this correlation remained when using a difference measure (difference of thresholds at 0.25 and 1.0 rpo, described above). In addition, the fact that some measures of spectral resolution were correlated with cognitive measures in the NH groups is a further indication that these behavioral measures of spectral resolution cannot be assumed to reflect solely peripheral processes (Neher *et al.*, 2012).

Overall, the relatively high proportion of variance accounted for in CI users by the non-auditory cognitive measures suggests that non-peripheral factors account for a significant proportion of the variance observed in speech perception across the population of CI users. This result mirrors recent findings in a study of NH young adults, which showed stable individual differences that generalized across three types of degraded speech: noise-vocoded speech, time-compressed speech, and speech in babble noise (Carbonell, 2017). It is possible that additional cognitive processes, such as inhibition-concentration (Moberly *et al.*, 2017) or cognitive control (Araneda *et al.*, 2015), in conjunction with working memory and non-verbal intelligence, may be influencing these strong correlations. Mean performance for CI users was also poorer than that of our NH groups on measures of both working memory and non-verbal intelligence, which raises questions about cognitive load and perhaps cognitive decline in CI users over time. Although the CI population is different from age-matched NH listeners in a number of ways, the results from this study cast some doubt on the notion that predominantly peripheral factors account for variability in the hearing outcomes of CI users.

### C. Vocoder fails to capture CI performance in NH listeners across auditory tasks

As a population, CI users come with a wide range of individual differences that are very difficult to control for in an experimental setting, including etiology of hearing loss, neural survival, duration of deafness, experience with hearing aids, exposure to American Sign Language, CI use in daily life, electrode placement, mapping, and more. The ability to simulate aspects of CI processing in NH listeners, therefore, provides an opportunity to study the contributions of implant processing, independently from these other factors (e.g., Bingabr *et al.*, 2008; Crew *et al.*, 2012; Dorman *et al.*, 1998; Fu and Nogaki, 2005; Grange *et al.*, 2017; Mesnildrey and Macherey, 2015; Shannon *et al.*, 1995). Consistent with several earlier studies (e.g., Henry and Turner, 2003; Litvak *et al.*, 2007; Oxenham and Kreft, 2014), our tone-excited vocoder with 12 dB/oct filter slopes to simulate current spread was successful in replicating average CI performance on the context sentences for both age-matched and young NH listeners. However, when the corpus of nonsense sentences was tested, performance between CI users and NH listeners was significantly different, with CI users' performance being much poorer.

It could be that our vocoder actually underestimated the degree to which spectral degradation influences speech perception in CI users, as indicated by performance in nonsense sentences. Arguing against this hypothesis is the fact that the CI users generally outperformed the NH listeners on the measures of spectral resolution, suggesting that (if anything) the CI users experienced less spectral degradation than the NH listeners. This apparent mismatch between poorer speech perception and better spectral resolution of CI users relative to age-matched NH listeners may be explained by the potential for non-uniform spectral resolution along the length of the cochlea in the CI users, caused perhaps by uneven neural survival and/or unequal electrode-neural interface quality across the electrode array. Any such unevenness would impact speech perception but may not affect our measures of spectral resolution, where performance could be based on the single cochlear location that provides the most information (Anderson *et al.*, 2011; O'Neill *et al.*, 2019).

The fact that the same vocoder was not able to match performance between task types, or even between different speech corpora, shows that the vocoder fails to capture some important aspects of CI perception that are currently not fully understood. It may be that simulating uneven spectral resolution across the frequency spectrum within a vocoder could potentially bring the measures of speech and spectral resolution in line.

### D. Limitations

One important conclusion that could be drawn from this study is that CI users rely more heavily on semantic context for speech understanding than NH listeners. However, before we accept this interpretation, other differences between the corpora should be considered. First, the sentences within each corpus had different structures, with the IEEE context sentences having five keywords per sentence, compared with the three keywords per sentence of the nonsense sentences. Second, the vocabulary was not matched between the corpora, with the words in the nonsense sentences being generally simpler than those used in the IEEE corpus. Third, each corpus was recorded using a different single female talker, meaning that idiosyncratic differences in intelligibility between the two talkers cannot be ruled out. Finally, our lack of counterbalancing of speech materials (having always presented context sentences first, followed by nonsense sentences) could have disproportionately affected performance on the nonsense sentences, if the degree of fatigue differed between groups. The future use of better-matched corpora, with similar vocabulary and sentence structure, spoken by the same talkers, and with counterbalanced presentation order could address these concerns. Finally, it may be that the absence of context changes the relative perceptual weights assigned to certain aspects of the speech (e.g., between consonants and vowels), which may in turn be represented differently by the vocoder and the actual CI.

When interpreting the apparently lower cognitive scores of the CI users, relative to the age-matched NH group, it should be noted that NH participants were recruited primarily by way of existing connections to the University of Minnesota, either as students, alumni, former faculty, or with a general interest or involvement in higher education. In contrast, CI users were recruited based solely on the basis of hearing history and current use of at least one CI. These different sampling strategies could have resulted in groups of NH listeners that were skewed towards higher cognitive function in relation to the general population. In addition, since the reading span test was verbal in nature, factors such as verbal fluency could have influenced working memory scores reported.

Finally, since the audiometric criteria used for our age-matched NH group was modified to facilitate close age-matching with older CI users, we cannot rule out distal effects of mild high-frequency hearing loss in our results (Yeend *et al.*, 2019). However, this appears unlikely as the average threshold for age-matched NH listeners at 8000 Hz was 22 dB HL and individual high-frequency thresholds did not correlate significantly with the vocoded speech perception scores.

## VIII. CONCLUSIONS

Both cognitive and peripheral factors that may influence speech perception in CI users were explored in 30 CI users, as well as in 30 age-matched and 30 young NH adults listening through a 16-channel vocoder that simulated substantial current spread, using filters with 12 dB/oct slopes. The main findings can be summarized as follows:

• CI users performed more poorly on sentences lacking semantic context than either NH group listening to degraded speech stimuli processed through a vocoder. This may indicate the importance of effective listening strategies gained over time, and a greater reliance of CI users on context to aid in speech understanding in everyday environments.
• Between-subject variance was greater for CI users than for either group of NH listeners in speech perception for speech stimuli both with and without context, as well as for some, but not all measures, of spectral resolution.
• Correlations involving measures of spectral resolution in NH listeners, even though resolution was limited by the vocoder, and not by individual differences, suggest that these measures of spectral resolution capture more than just peripheral contributions to perception.
• Significant correlations in CI users and age-matched NH listeners between measures of speech perception and cognitive factors highlight the influence of non-peripheral factors in understanding degraded speech.
• The vocoder used to process auditory stimuli for both groups of NH listeners accurately reflected performance in CI users for context sentences, but not for nonsense sentences or for measures of spectral resolution. This result suggests that current vocoders fail to capture important aspects of CI performance.

## Footnotes

^1^ Current URL: https://ubiq-x.gitlab.io/rspan/.
